# Depression Symptom Trajectories and Associated Risk Factors among Adolescents in Chile 

**DOI:** 10.1371/journal.pone.0078323

**Published:** 2013-10-11

**Authors:** Lexine A. Stapinski, Alan A. Montgomery, Jon Heron, John Jerrim, Anna Vignoles, Ricardo Araya

**Affiliations:** 1 School of Social and Community Medicine, University of Bristol, Bristol, United Kingdom; 2 Institute of Education, University of London, London, United Kingdom; 3 Faculty of Education, University of Cambridge, Cambridge, United Kingdom; University of Akron, United States of America

## Abstract

Adolescence is a key period for studying the development of depression, with studies in Europe and North America showing a pattern of elevated risk that begins in early adolescence and continues to increase as adolescents age. Few studies have examined the course of adolescent depression and associated risk factors in low and middle-income countries. This longitudinal cohort study examined depression symptom trajectories and risk factors in a sample of socio-economically disadvantaged adolescents in Chile (*n* = 2,508). Data were collected over an 18-month period as part of a clinical trial for secondary students aged 12 to 18 (median age 14). Clinical levels of depression were prevalent in this sample at baseline (35% for girls and 28% for boys); yet latent growth models of symptom trajectories revealed a pattern of decreasing symptoms over time. There was evidence of an anxiety-depression developmental pathway for girls, with elevated anxiety levels initially predicting poorer depression outcomes later on. Poor problem-solving skills were associated with initial depression levels but did not predict the course of depressive symptoms. Critically, the declining symptom trajectories raise important methodological issues regarding the effects of repeated assessment in longitudinal studies.

## Introduction

### Adolescent Depression

Depression in adolescence is a serious public health issue, with concerning developmental and psychosocial consequences including increased risk of suicide [1,2]. Adolescent depression tends to run a relapsing course persisting well into adulthood [3], and is associated with long-term impairments in occupational performance, interpersonal functioning, physical health and quality of life [4]. Notably, adolescent depression is associated with reduced life satisfaction in adulthood even after adjusting for confounding socio-demographic factors and concurrent psychopathology, suggesting that adolescents are permanently changed or scarred by the experience [4]. Given the chronic recurring course and negative sequelae that can be associated with adolescent depression, effective preventative and early intervention strategies are important.

Depression is a highly prevalent and disabling condition in Chile, where it is ranked the 2^nd^ most common cause of disease burden [5]. Comparison of adulthood prevalence rates suggest that depression is more common in Chile than in the UK or other South American countries [6,7], which has been attributed to the considerable changes which have occurred to the socio-political landscape and lifestyle in Chile [8]. To our knowledge, only one study has specifically examined prevalence rates in Chilean children and adolescents; the results indicated higher 12-month prevalence of depression in adolescents aged 12 to 18 (7.0%) as compared to younger children (3.5%) [9]. This pattern is consistent with studies conducted in Europe and North America which show a period of elevated risk for depression that begins in early adolescence and continues to increase as adolescents age [10-14]. The rise in depression during adolescence has been attributed to biological and social-contextual changes that accompany this period of development, such as pubertal changes, increased stress levels, maturation of brain circuitry and enhanced social and self-awareness [15]. Adolescence also marks the emergence of gender differences in the prevalence of depressive symptoms, with higher prevalence observed among girls, a finding that is consistent across countries including Chile [16]. 

### Risk Factors for Depression

Understanding the risk factors for adolescent depression is an important first step for developing strategies to reduce the severity, chronicity, and long-term impact of this disorder. Prior research has identified risk factors within 3 broad categories: i) individual adolescent factors, encompassing characteristics such as gender, temperament and coping skills; ii) family factors, such as parent psychopathology and parenting style; and iii) social-contextual factors, such as life stressors and friendship quality (see reviews in [1,17]). To our knowledge, risk factors for adolescent depression are yet to be examined in a Chilean sample. In this paper we focus on individual factors that may predict susceptibility to depression for Chilean adolescents. 

A growing body of research conducted in Europe and North America suggests that symptoms of anxiety are important developmental precursors to depression. Anxiety and depression are frequently comorbid during adolescence, yet the onset of anxiety symptoms typically precedes depression [18-20]. Furthermore, pre-existing anxiety appears to reliably predict the onset and exacerbation of depression symptoms [21-23]. This has led to the suggestion that anxiety and depression may be different expressions of the same underlying disorder, with anxiety tending to transition to depression as an adolescent matures [24,25]. Contrary to this view, however, a recent structural equations analysis indicated these disorders are best represented by two parallel but distinct developmental trajectories [26]. The link between anxiety symptoms and subsequent depression may be explained by shared genetic or environmental vulnerabilities, with evidence from twin-studies suggesting a common set of genes and environmental factors that influence both disorders [27,28]. Others have argued that pre-existing anxiety *directly* influences an adolescent’s vulnerability to depression, perhaps via the concomitant increase in negative affectivity or impairment to social and academic functioning [21,22]. Supporting this perspective, Woodward and Fergusson [29] provided evidence that the relationship between anxiety symptoms and subsequent depression was independent of a comprehensive set of common familial and environmental vulnerabilities, including IQ, maternal education, family functioning and socioeconomic status. Moreover, several authors have suggested that the interplay between anxiety and depressive symptoms may account for the gender differences in depression that emerge during adolescence [25,30,31]. Specifically, girls may be susceptible to the development of depression due to higher incidence of childhood anxiety, or greater propensity to become depressed following an anxiety disorder. Researchers have emphasized the need for longitudinal research to clarify the gender differences, temporal sequence and nature of the relationship between anxiety and adolescent depression [29,31]. 

While some individual factors increase risk, other characteristics may protect against the development of depressive symptoms [32]. In particular, the capacity for effective problem-solving has been identified as a protective coping skill that helps to minimize the psychological consequences of stress and adverse life circumstances [33-36]. Effective problem-solving is defined as the systematic and skilful application of problem-solving principles, encompassing problem definition and formulation, generation of alternative solutions, decision making, solution implementation and verification [37]. Adaptive social problem-solving skills may be especially relevant during adolescence, as teens navigate changing peer relationships and the increasing importance of peer approval [38]. Indeed, the few studies to investigate this relationship prospectively have confirmed an association between poor social problem-solving skills and the development of adolescent depression [32,39]. 

### The Current Study

To date, most research investigating adolescent depression has been conducted in Western countries, although there is increasing acknowledgement of the need to extend this research to more diverse populations [15]. We previously conducted a randomized controlled trial to evaluate the effectiveness of a depression prevention program implemented in low-income schools in Chile. In brief, the main trial analyses showed no benefit in depression symptoms for those adolescents receiving the prevention program [40]. In view of this null effect, the dataset was used for the present analysis. This paper extends our earlier report by adopting latent growth analysis to explicitly model individual depression trajectories over time within this cohort. By examining between-individual variation in symptom trajectories, we aim to identify factors that place adolescents at risk for persistent or recurring depression. A related aim is to address gaps in the literature by exploring dynamic associations between time-varying anxiety symptoms and exacerbation of depression over time. Understanding the risk factors that are relevant in this group, and how this differs from the predominantly middle-income Western samples examined previously, will inform the refinement of future prevention programs and translation to the Chilean context. 

We specified a number of hypotheses a priori based on previous research findings. Our first was that depression symptoms would increase with age, with more marked symptoms evident for girls as compared to boys. Second, we predicted that problem-solving ability would play a protective role, predicting slower rate of change in depression over time. Third, we hypothesised that initial levels of anxiety would predict an exacerbation of depression symptoms over the course of the study. Gender differences in the association between depression and anxiety were examined to evaluate the hypothesis that girls would be more susceptible to the anxiety-depression developmental pathway. 

## Method

Data for this study were collected between 2009 and 2011 as part of a randomised controlled trial of a prevention program for depression; the full trial protocol, sample size calculation and main analyses have been published elsewhere [40,41]. These analyses revealed no difference in depression symptoms at 6 and 18 months post randomization between participants receiving the intervention and those in the no-intervention control group. Nevertheless, we included trial arm in all analyses to adjust for any residual effect on psychological symptoms. Requests for access to raw data for this study can be directed to the last author (R.Araya@bristol.ac.uk). 

### Ethics Statement

The consent process for this study involved three stages. We followed procedures that had received ethics approval for similar research implemented in secondary schools in the United Kingdom [42,43]. Firstly, eligible schools were provided with information about the study, and interested head teachers were required to provide written confirmation that their school wanted to participate. Schools agreeing to participate included the intervention program as part of their curriculum; approval to do so was obtained from the Ministry of Education. Secondly, all eligible adolescents were provided with an information letter to give to their parents; this letter was also posted to parents at their home address. Parents were invited to return a written form opting out of the study assessments if they did not wish their child to participate. They were advised that they could withdraw their child from the study at any time. An opt-out parental consent procedure was approved by the ethics committee due to the minimal risk and potential benefits associated with participation in this study. Finally, adolescents were provided with information about the study and signed a written consent form if they decided to opt in. Data were destroyed for students whose parents expressed their option for withdrawal from the study. The study procedures were reviewed and approved by the Ethics Committee of the Clinical Hospital, Faculty of Medicine, Universidad de Chile, and the Chilean Ministries of Health and Education. 

### Setting and Participants

Twenty-two schools were randomly selected for participation from a sampling frame comprising all municipal secondary mixed-sex schools with two or more '1° Medio' classes (equivalent to 9th grade) in Santiago, Chile. Municipal schools provide education for most low-income, secondary school students in Santiago. All students attending '1° Medio' grade in the selected schools were invited to participate. The majority of students (78%) in this grade were aged 14 or 15 years; however due to inter-individual differences in school progression the full age range for the current sampe was 12 to 18 years. Adolescents reporting severe depressive episodes and/or clear suicidal risk were encouraged to seek professional advice. Once recruitment was finalised, schools were randomly allocated to the intervention or no-intervention control condition. The intervention was based on a cognitive behavioural therapy model, and comprised 11 weekly and 2 booster sessions each lasting approximately one hour (for a full description, see 41). The control group received standard school curriculum with no input from the research team. 

Within the 22 schools sampled, all except 4 students consented to participate; in total, 2,508 adolescents were enrolled in the study at baseline. Depression and anxiety data were available for 99.9% (*n* =2,505) of participants at baseline, 82% (*n* =2,061) of participants at the 6 month assessment, and 77% (*n* =1934) of participants at the 18 month assessment. An additional 22 participants were missing data on one or more baseline covariate measures. Participants with complete outcome and covariate data had less severe depression at baseline (*M* = 13.0, 95% CI: 12.5 to 13.5) as compared to those with partial data (*M* = 14.4, 95% CI: 13.7 to 15.2). Baseline anxiety did not differ as a function of attrition status, but participants with complete data reported better problem-solving skills (*M* = 45.4, 95% CI: 44.8 to 46.0) compared to those with partial data (*M* = 43.3, 95% CI: 42.4 to 44.2). Participants with complete data were also more likely to be female, χ^2^(1) = 12.0, *p* = 0.001, and younger (*M* = 14.4, 95% CI: 14.36 to 14.43) than those with partial data (*M* = 14.8, 95% CI: 14.8 to 14.9).

### Measures

Participants completed demographic questions and the following self-report measures at baseline, and again 6 and 18 months later. 

#### Beck Depression Inventory II (BDI-II)

Depression symptoms were assessed using the adolescent version of the BDI-II [44]. This brief and well-established measure has been translated to several languages including Spanish and demonstrates good psychometric properties when used among adolescents in Chile [45]. Further criterion validation of this scale against a clinical interview was performed within a subsample of adolescents to establish cut-off scores according to a clinical diagnosis of depression [46]. Although the optimal cut-off point for the whole sample was ≥17, there were important differences between genders, with the cut-off point for boys (≥14) being much lower than for girls (≥20). There were no clear differences in cut-off points according to age.

#### Revised Child Anxiety and Depression Scale (RCADS)

Adapted from the Spence Child Anxiety Scale [47], this continuous measure for youth is designed to assess symptoms corresponding to DSM-IV clinical syndromes. In this study we used a reduced 15-item Spanish language version of the scale which has demonstrated good factorial validity, internal consistency and construct validity within a community adolescent sample [48]. The full scale consists of 6 subscales, each comprising 5 items, however we administered only the 3 anxiety subscales considered most relevant for the sample (social phobia, generalised anxiety disorder, panic disorder). 

#### Social Problem-Solving Inventory Revised (SPSI-R)

We administered the Rationale Problem-Solving subscale of the SPSI-R [37], which comprises 20 items assessing constructive problem solving across 4 domains: problem definition and formulation, generation of alternative solutions, decision making, solution implementation and verification. Total scores provide an index of global problem-solving skills, with higher scores indicating greater ability. The Spanish adaptation of this measure has demonstrated sound psychometric properties [49], and is suitable for use in the school context [50]. 

### Data Scoring and Analysis

#### Data Preparation and Preliminary Analyses

Unanswered items from a multi-item scale were replaced with the mean of the participants’ own responses to the rest of the scale items provided they had valid responses for at least 80% of the items. Descriptive statistics and plots obtained using STATA (release 12.0) software were used to characterise the sample and check for univariable and multivariable non-normality. Adolescents were recruited to the study within school groups, however preliminary multi-level analyses in STATA indicated minimal between-school variation for depression trajectories, thus subsequent analyses adopted more parsimonious one-level models. 

 Latent growth modelling. *Latent growth modelling is a statistical method ideally suited for examination of individual differences in change over time (for a detailed introduction, see 51*)*. Within this framework, change over time is estimated using two unobserved latent factors: i*)* the first defines the growth intercept, and represents initial levels at the starting point of the growth curve; ii*)* the second defines the slope of the growth curve and represents the rate of change over time. We fit a series of linear growth models using Mplus 5.2 software [52] to examine depression trajectories and explore factors associated with individual variation in change over time. As non-normal distribution was indicated for the anxiety and depression variables, all models were estimated using maximum likelihood with robust standard errors (MLR*)* which is robust to violations of multivariate normality.*

As a first step, depression trajectories over time were modelled within an unconditional linear growth model; this term refers to a basic model with no predictors included. Factor loadings for the intercept factor were set to 1.0 to reflect the initial status, while loadings for the slope factor were fixed to represent linear change over time. An advantage of latent growth modelling is the flexibility it affords in specifying the error variances and covariances of these repeated measurements [53]. We compared models with homoscedastic and freely estimated error variances in order to identify the most appropriate residual structure for the data. Once an appropriate base model was established, participant age and gender were added to examine the relationship between these individual characteristics and depression growth rates. There were small numbers in some age groups, thus data were collapsed into 3 age categories: i) age 14 and younger (*n* = 1410); ii) age 15 (*n* = 690); iii) age 16 and older (*n* = 387). Trial allocation was also included to adjust for any residual impact on symptom change over time. 

Unlike traditional longitudinal approaches which are limited to the investigation of static baseline risk factors, latent growth modelling allows for inclusion of time-varying predictors, so that parallel changes in outcome and predictors can be modelled [54]. We used the parallel growth methodology recommended by Curran et al. [51] to concurrently model anxiety and depression trajectories and investigate the relationship between the two sets of growth parameters. Initially, an unconditional growth model was fit individually for time-varying anxiety; next growth curves for depression and anxiety were estimated simultaneously. Residual variances of the repeated measurements on each occasion were allowed to covary between constructs to accommodate common method variance at each assessment. Covariances were estimated between the anxiety and depression intercept factors, and the anxiety and depression slope factors. In addition, the depression and anxiety slopes were each regressed on the intercept factors of the growth curves. This allowed examination of the relationship between initial levels of anxiety and change in depression, adjusted for initial depression levels. The impact of age and trial allocation on the growth factors of both time-varying variables was also adjusted for. In this final model, baseline problem-solving ability was included to examine the potentially protective effect on depression trajectories. Gender differences were examined using a multiple group framework. First, a baseline model was developed that estimated all parameters separately for boys and girls, with the exception of residual variances for observed variables which were constrained to be equal because measurement invariance can bias group comparisons of parameter estimates. Next, increasingly restrictive equality constraints were imposed, and gender differences were evaluated using Satorra-Bentler adjusted chi-square difference tests calculated relative to the baseline model [55].

#### Missing Data & Model Evaluation

Participants with partial data were included in all latent growth models; data were assumed to be missing at random (MAR) conditional on the observed covariates and outcomes. Under this assumption, missing data is accommodated through full-information maximum likelihood which estimates parameters using all available data [52]. Acceptability of each growth model was evaluated using standard goodness of fit indices. The chi-square test of exact fit is stringent and sensitive to sample size with simulations showing the test will routinely reject good models when sample size is large (e.g., n > 200) [56-58]. Thus we also considered several relative fit indices designed to avoid problems associated with relying solely on the chi-square test. A root mean square error of approximation (RMSEA) value less than 0.06, standardized rootmean-square residual (SRMR) index less than 0.08, Tucker-Lewis index (TLI) and comparative fit index (CFI) values greater than 0.95 are considered indicative of good fit to the data [59]. 

#### Sensitivity Analyses

The primary analyses revealed a pattern of results that was unexpected based on the extant literature, thus we conducted a series of sensitivity analyses to confirm the accuracy of the results. Given the large age range of adolescents in the study (12 to 18), we repeated our growth analyses within a more restricted sample of adolescents aged 14 to 15 (78% of the sample). Supplementary analyses in STATA and MPlus were used to examine whether decreasing symptom trajectories were attributable to response attrition, a small number of extreme responders, effects of the intervention, or skewed data distribution. We also explored whether the observed pattern of results might be attributable to the statistical artefact regression to the mean. Mean change in depression was plotted for each decile of baseline depression scores to examine whether greater change was evident for scores at the tails of the distribution. We then applied a variant of the covariance-based Mee-Chua test [60] that purports to distinguish between regression to the mean effects and true change. While the original Mee-Chua test assumes a known population mean (µ), the revised test uses differential calculus to systematically vary the population mean over a range of reasonable values. We implemented this test using the Excel template provided by Ostermann et al. [60] to examine change from baseline to 18 month follow up using square root transformed data given the test assumes a normal distribution. 

## Results

### Sample Characteristics & Depression Prevalence

The sample comprised 2508 adolescents, of which there were marginally more males than females. The mean age for the whole sample at baseline was 14.5 years with a range from 12 to 18 years but with 78% aged 14 or 15 years old. Table 1 presents means and standard deviations for all predictor and outcome variables. At baseline, the prevalence of likely clinical depressive disorder using gender-specific cut-off points was 35% and 28%, among girls and boys respectively. 

**Table 1:** Means (standard deviations) of main variables overall and by gender.

**Variable**	**Girls**	**Boys**	**Overall Sample**
*n* (*%*)	1115 (*44.5%*)	1393 (*55.5%*)	2508
Age	14.5 (*0.9*)	14.6 (*0.9*)	14.5 (*0.9*)
Problem-solving ability	44.8 (*12.2*)	44.8 (*13.3*)	44.8 (*12.8*)
Time 1: Depression	16.8 (*11.3*)	10.8 (*8.4*)	13.4 (*10.2*)
Time 2: Depression	15.6 (*11.2*)	9.5 (*8.4*)	12.3 (*10.3*)
Time 3: Depression	12.3 (*11.3*)	7.8 (*8.4*)	9.9 (*10.1*)
Time 1: Anxiety	22.1 (*8.7*)	17.8 (*7.9*)	19.7 (*8.5*)
Time 2: Anxiety	21.1 (*9.0*)	16.7 (*8.4*)	18.7 (*8.9*)
Time 3: Anxiety	17.8 (*10.3*)	14.2 (*8.9*)	15.9 (*9.7*)

### Latent growth models

#### Unconditional Growth Models

**Figure 1:**
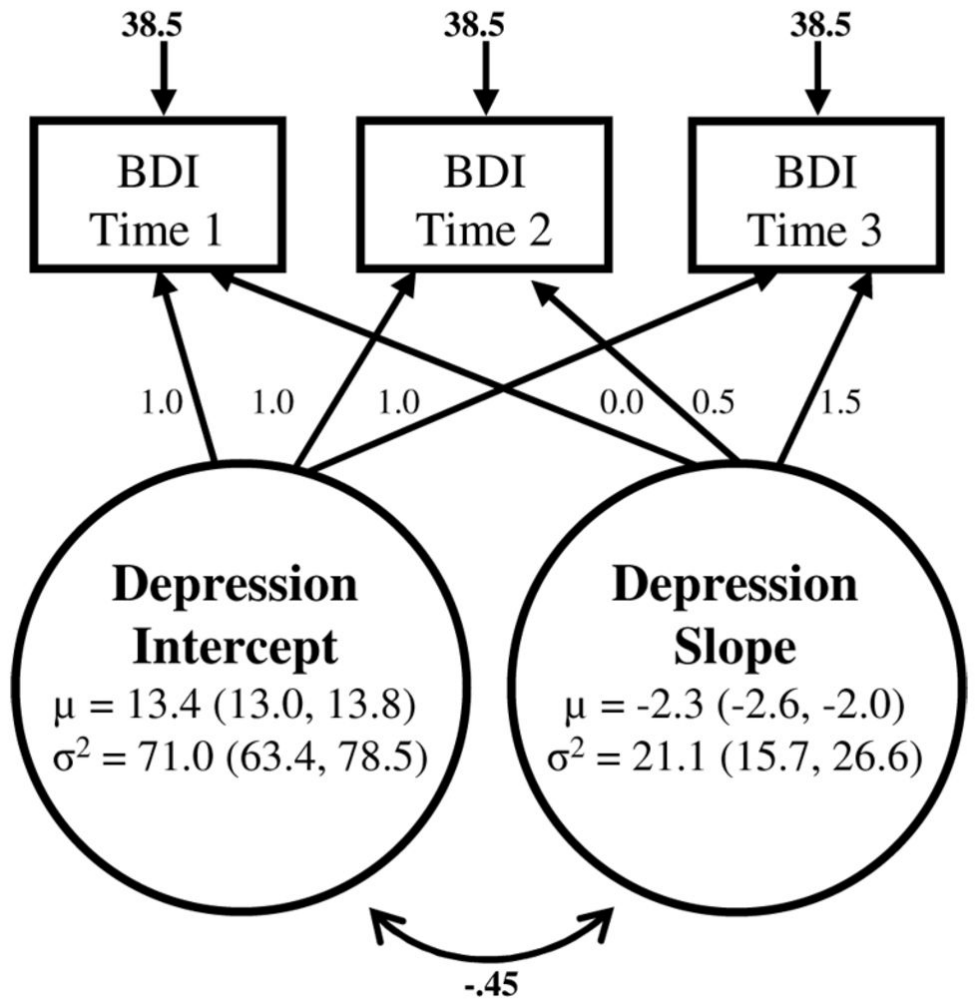
Unconditional growth model for adolescent depression. µ indicates model estimated mean, σ^2^ indicates estimated variance, with 95% confidence intervals in parentheses. Fit indices for the homoscedastic model were χ^2^(3) = 11.01, *p* = 0.01; TFI = 0.99; CFI = 0.99; RMSEA = 0.03 (95% CI: 0.01 to 0.05); SRMR = 0.04. All estimates are reported in raw score units.

Model specification and fit indices for the unconditional linear growth model of depression are shown in Figure 1. Although the freely estimated residual structure was preferred based on the chi square test of exact fit, relative fit indices suggested good model fit for the homoscedastic and freely estimated residual structure. The pattern of results was similar for both models; thus in the interests of parsimony we retained the homoscedastic error structure for this and all subsequent models (parameter estimates for this model are shown in Figure 1). A negative mean value for the slope factor indicated that the overall group reported decreases of 2.3 points on the BDI-II per year (*p* < 0.001). However, individual variability was indicated for both growth factors. A moderate negative correlation between the intercept and slope factors (*r* = -0.48; *p* < 0.001) indicated that adolescents with higher depression scores initially tended to experience a steeper decline in symptoms. 

An individual growth model was also estimated for time-varying anxiety. Again we retained the parsimonious homoscedastic error structure as relative fit indices suggested adequate fit to the data ( χ^2^(3) = 21.42, *p* < 0.001; TFI = 0.98; CFI = 0.98; RMSEA = 0.05, (90% CI: 0.03 to 0.07); SRMR = 0.05). Showing a similar pattern to depression, the slope mean indicated decreasing anxiety (µ = -2.6; 95% CI: -2.9 to -2.3) over time, and an inverse relationship between initial status and change over time (*r* = -0.29; *p* < 0.001). 

#### *Gender & Age Effects.*

**Figure 2:**
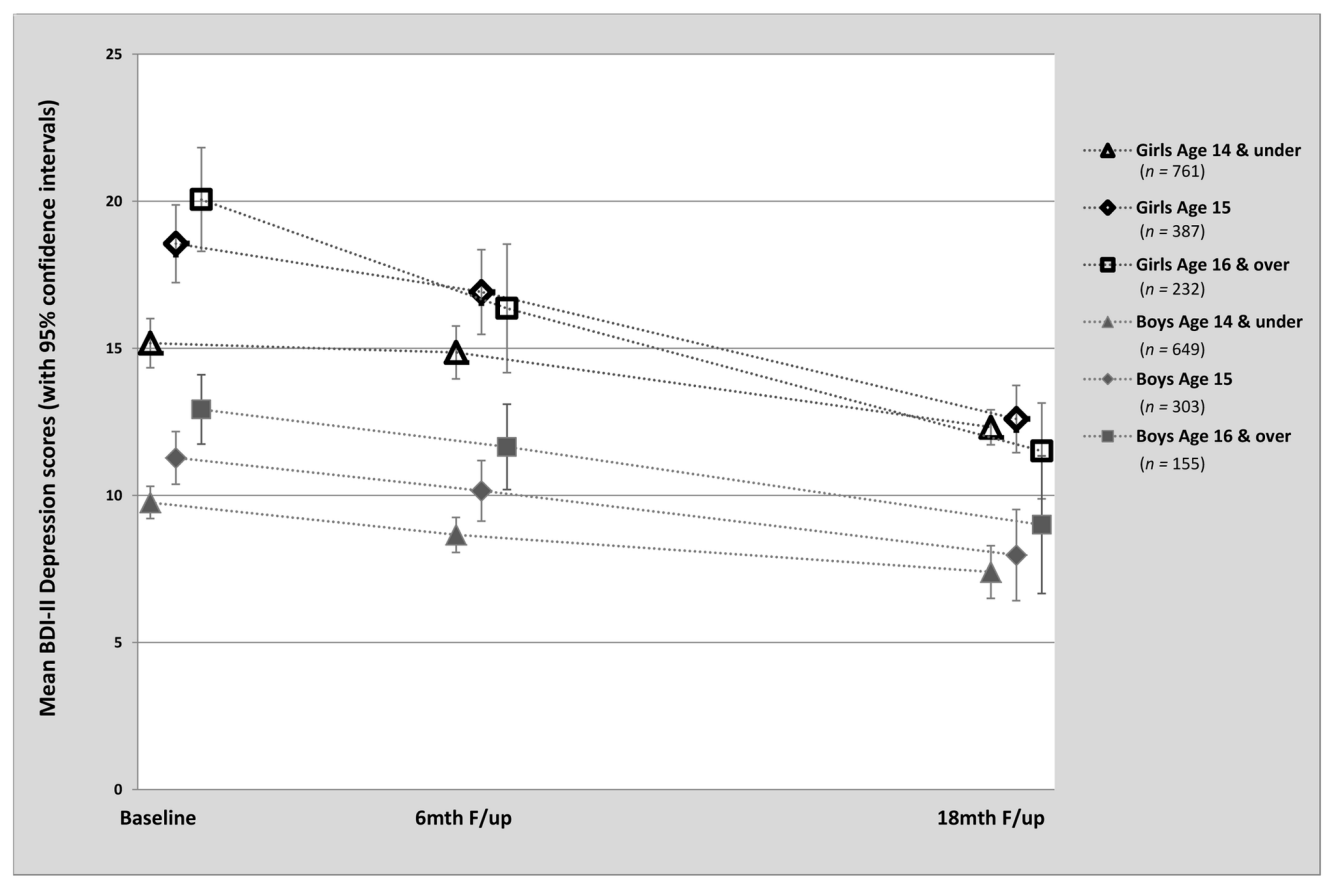
Mean Depression Scores by Gender and Age group at Baseline, 6 month and 18 month follow-up. Note: Age data were missing for 21 participants.

Table 2 shows model fit and parameter estimates when gender and age were added to the depression growth model. Relative fit indices suggested good fit to the data. Decreased variance estimates for both growth parameters suggested inclusion of these variables accounted for some, but not all, of the individual variability in the data. Regression estimates suggested a positive association between the intercept factor and participants’ age (*p* < 0.001) and gender (*p* < 0.001), indicating that initial levels of depression were higher for females, and older adolescents. In contrast, an inverse relationship was observed between the depression slope and participants’ age (*p* < 0.001) and gender (*p* = 0.007), indicating that older adolescents and girls improved more rapidly over time. Figure 2 illustrates this relationship by showing mean depression scores over time by gender and age group. Consistent with the main trial analyses [40], there was no evidence of an effect of trial allocation on the depression slope.

**Table 2:** Estimates and Confidence Intervals for Conditional Depression Growth Models.

**Model**	**Variable**	**Parameter Estimates for Predictors on Latent Factors *B* ( 95% CI)**			
		**Depression Intercept**	**Depression Slope**	**Anxiety Intercept**	**Anxiety Slope**
***Model 1: Age & Gender***					
	Gender	6. 35 (5.58 to 7.12)	- 0.89 (-1.54 to -0.24)	..	..
	Age	1. 94 (1.44 to 2.44)	- 1.15 (-1.62 to -0.68)	..	..
	Trial Allocation	..	0.27 (-0.28 to 0.81)	..	..
***Model2:Problem-solving&Anxiety***					
	Age	1.79 (1.31 to 2.27)	-0.64 (-1.06 to -0.22)	0.94 (0.51 to 1.36)	-0.90 (-1.33 to -0.47)
	Trial Allocation	..	0.28 (-0.24 to 0.79)	..	0.66 (0.14 to 1.18)
	Problem-Solving	G: -0.21 (-0.26 to -0.15)	0.01 (-0.02 to 0.04)	0.07 (0.04 to 0.10)	-0.04 (-0.07 to -0.01)
		B: -0.11 (-0.14 to -0.07)			
	Depression Intercept	..	G: -0.35 (-0.44 to -0.26)	..	-0.02 (-0.09 to 0.06)
			B: -0.18 (-0.32 to -0.04)		
	Anxiety Intercept	..	G: 0.17 (0.08 to 0.26)	..	-0.15 (-0.24 to -0.05)
			B: 0.05 (-0.06 to 0.12)		

Note. All estimates are reported in raw score units. For each estimate 95% confidence intervals are presented in parentheses unless otherwise stated. Parameter estimates are shown separately for girls (G) and boys (B) when gender differences were indicated by nested chi-square difference tests.

Fit indices for Model 1: χ^2^(7) = 16.4, *p* = 0.02; TFI = 0.99; CFI = 0.99; RMSEA = 0.02 (90% CI: 0.01 to 0.04); SRMR = 0.02.

Fit indices for Model 2: χ^2^(53) = 128.7, *p* < 0.001; TFI = 0.97; CFI = 0.98; RMSEA = 0.03 (90% CI: 0.03 to 0.04); SRMR = 0.03.

#### Risk Factors: Anxiety & Problem-solving ability

**Figure 3:**
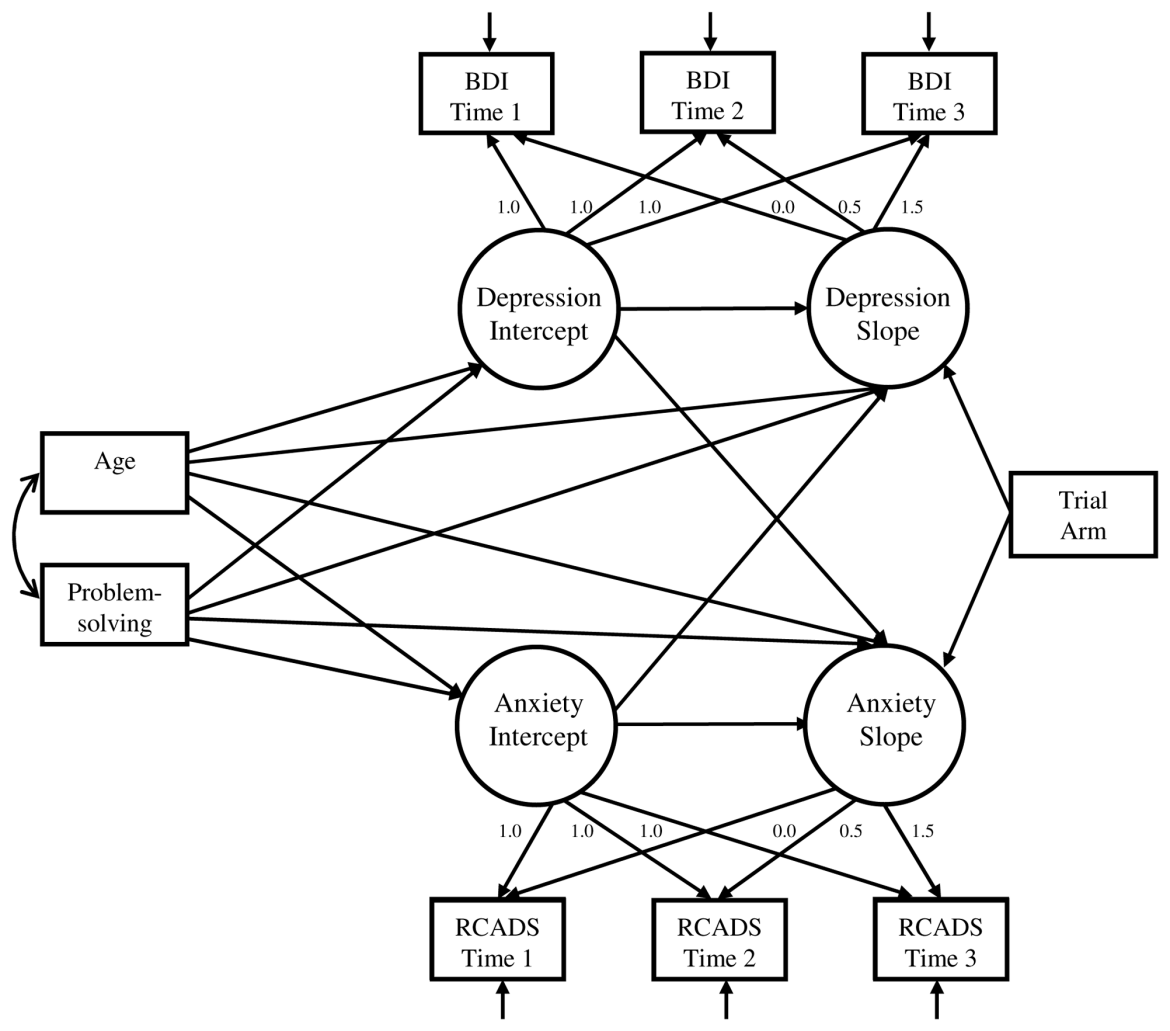
Model specification for the full growth model of adolescent depression. A multi-group framework was used to model gender differences on parameter estimates. To reduce figure complexity residual covariances are not shown. Residual variances of the repeated measurements on each occasion were allowed to covary, and covariances were estimated between the anxiety and depression intercept factors, and the anxiety and depression slope factors.

Model specification for the full model incorporating problem-solving and time-varying anxiety is depicted in Figure 3. In view of the finding of gender differences in depression trajectories, a multiple group framework was used. Comparison of model fit indices confirmed superior model fit when parameter estimates were allowed to vary by gender, compared to a more restricted model with parameters constrained to be equal (χ^2^(32) = 504.9, *p* < 0.001). Difference tests comparing increasingly restrictive equality constraints to the freely estimated baseline model suggested gender differences for 3 parameter estimates and the latent factor variances. Girls were characterised by greater residual variances for the growth factors as compared to boys. Estimates, 95% confidence intervals and model fit indices for the final model are shown in Table 2. Gender differences were observed for the effect of initial severity on depression trajectories, with this effect more prominent for girls. 

Of primary interest was the relationship between anxiety, problem-solving skills and change in depression over time. Large correlations between latent slope factors suggested that anxiety and depression decreased in parallel for girls (*r* = 0.61) and boys (*r* = 0.54). A positive relationship between anxiety intercept and depression slope was observed for girls (*p* < 0.001), but not boys (*p* = 0.39). For girls, this result suggests that higher initial anxiety predicted less improvement in depression symptoms over time. The reverse relationship between initial levels of depression and subsequent anxiety change was not observed for either gender. To explore the magnitude of the relationship between initial anxiety levels and change in depression for girls, we calculated the difference in estimated slope for female participants with relatively low and high initial anxiety levels. An increase of one standard deviation (6.79) on the anxiety intercept variable was associated with a 1.15 BDI-II unit change on depression slope. The overall depression slope for girls was estimated at -2.96 BDI-II units per year; at higher levels of initial anxiety the slope was estimated at -1.81 BDI-II units per year, and at lower levels of initial anxiety there was a steeper slope of -4.11 BDI-II units per year. 

Baseline problem-solving ability was negatively associated with initial depression level for both genders (girls: *p* < 0.001; boys: *p* < 0.001), although the size of effect was greater for girls. To explore the magnitude of this relationship we calculated the difference in estimated depression level for participants with relatively low and high initial problem-solving ability. An increase of one standard deviation on initial problem-solving ability was associated with an estimated decrease in initial depression levels of 1.42 BDI-II units for boys, and 2.51 BDI-II units for girls. Contrary to hypotheses, problem-solving ability was not related to change in depression symptoms (*p* = 0.40). There was an unexpected positive association between problem-solving ability and initial anxiety levels (*p* < 0.001), and a modest negative relationship between problem-solving ability and rate of anxiety change (*p* = 0.008). 

### Sensitivity Analyses

Although the pattern of declining symptom trajectories over time was contrary to expectation, a comprehensive series of sensitivity analyses confirmed the robustness of these results (full data are not reported but will be made available on request). There was no material difference to the pattern of results when growth analyses were restricted to adolescents aged 14 to 15 only. The pattern of data in Figure 2 was reproduced when square root transformed and median scores were plotted, suggesting the declining symptom trajectories were not attributable to skewed data distribution or the influence of a small number of extreme scorers. No material difference to the pattern of results was observed when exploratory and growth analyses were repeated using complete case data or data from the control group only. Plots of mean depression change within deciles of baseline depression scores revealed a pattern of descending difference scores for the top 7 deciles, but stable or increasing scores in the bottom 3 deciles. However, the *p*-values obtained from the Mee-Chua test for regression to the mean [60] showed evidence of real change (i.e independent of regression to the mean effects) when the true population could be assumed to lie below a BDI-II score of 4.8 or above a score of 6.0. Given that norms within non-clinical samples suggest a mean score of around 12.5 [61], or higher in a Chilean sample [62], it seems improbable that the true mean in our target population is less than 6. Therefore, it is unlikely that the observed decline in depression scores can be attributed entirely to regression to the mean. 

## Discussion

This study examined depression trajectories and risk factors within an under-researched sample of socio-economically deprived adolescents in Chile. Within this sample the prevalence of likely clinical depression at baseline was 35% for girls and 28% for boys, figures considerably higher than those typically observed in high-income western countries [63,64] . Investigation of longitudinal symptom trajectories revealed four main findings. First, contrary to previous epidemiological findings there was no evidence for increasing depressive symptoms with increasing age. Instead, our findings showed an overall growth pattern of decreasing symptoms over time. Second, whilst there was evidence of gender differences in depression trajectories, these were in the opposite direction to expected, with symptoms declining more rapidly for girls as compared to boys. Third, we observed that initial anxiety levels were predictive of slower depression recovery for girls but not for boys. Finally, we found that problem solving skills were associated with initial depression levels but did not predict the course of depressive symptoms. As will be elaborated further, these findings have important implications for both clinical practice and research methods. 

### Depression Symptom Trajectories

Epidemiological studies consistently show a pattern of increasing depression symptoms between early to late adolescence [10-14]. We replicated this pattern cross-sectionally, finding that older adolescents reported more severe depressive symptoms at the baseline assessment. However, the longitudinal data showed a contrasting pattern of decreasing symptoms on repeated measurements as adolescents became older. On average, adolescents within this cohort reported a decrease of 2.3 points (equivalent to 0.22 of a standard deviation) on the BDI-II per year. To put this into context, this decrease is comparable in size to the mean change on the BDI-II from baseline reported in a previous study for adolescents receiving a school-based prevention program [65]. However, in our study the observed symptom reduction was independent of any intervention effects, as the main trial analyses excluded any meaningful effect of the intervention [40], and all analyses were adjusted for trial allocation but this variable had no impact on depression outcomes. It is important to note that we explored this unexpected pattern of results with a comprehensive set of sensitivity analyses, all of which confirmed the pattern of results. Although age predicted more severe depression initially, older adolescents reported a greater decrease in symptoms over time. Similarly, female gender predicted more severe depression levels at baseline, but was associated with a more rapid reduction in symptoms over time as compared to boys. The resounding pattern was that individuals with more severe initial depressive symptoms experienced the steepest recovery. 

Although this decrease in depression symptoms over time was not expected based on the epidemiological literature, we note that a number of other large trials have observed a similar decrease in depression symptoms for participants receiving no intervention [65-67]. There are a number of possible explanations for these declining symptom trajectories. Firstly, the decline in symptoms may reflect the fluctuating course of depression, with symptomatic adolescents reporting a natural alleviation of symptoms by subsequent assessment. However, this explanation does not adequately account for the observed pattern of results, as sensitivity analyses indicated declining symptom trajectories within the overall sample that were not driven solely by participants with the highest symptoms at baseline. A related explanation is regression to the mean, a statistical artefact that occurs when repeated measurements are taken from the same individual over time. As each measurement incorporates a component of random measurement error, relatively low or high initial scores are likely to be followed by less extreme scores closer to the individual’s true mean [68,69]. As a result, natural variation in repeated observations can be mistaken for real change [70]. Although traditional accounts indicate that regression to the mean effects would be expected at a second measurement, but not subsequently, it has been demonstrated that a decreasing sequence of error autocorrelations (as commonly seen in developmental data) would lead to continuing regression to the mean with each repeated measurement [71]. Regression to the mean is commonly discussed in reference to studies where groups are selected based on extreme scores, although it is not limited to this context [69]. There is a lack of consensus within the field as to the best approach for quantifying or adjusting for possible regression to the mean, and indeed some authors argue that this phenomenon has been dramatically overstated [72,73]. We selected the adjustment technique best suited to our study design [60], and based on this test concluded that the observed decline in depression symptoms was unlikely to be accounted for entirely by regression to the mean effects. 

A second possible explanation for the depression trajectory we observed is response bias associated with repeated testing. Retesting may alter or bias the way that participants respond to items on subsequent measurement occasions in a number of ways. Prior exposure to the questions may improve understanding of the meaning of the survey questions, resulting in more reliable responses at a later assessment [74]. On the other hand, less reliable responses may be observed at subsequent assessment because participants are motivated to present themselves more favourably (i.e., socially desirable responding) [75], or have gained a better understanding of the burden associated with item responses (e.g., answering yes may lead to extra questions or unwanted attention) [74]. The possibility of a testing effect when measures of negative mood states are repeatedly administered has been noted previously [75,76]. Over 6 waves of assessment within an adolescent sample, Twenge and Nolen-Hoeksema [76] found a pattern of steadily decreasing depressive symptoms which they attributed to habituation to the item content of the Children’s Depression Inventory. Some authors have suggested that diagnostic interview may be less susceptible to retesting effects than self-report measures [63,76]. This may explain the discrepancy between our results and longitudinal cohort studies using clinical interview [11,12,63], but not other studies finding increasing depression over the course of adolescence using repeated self-report measurements [10,13,14].

A third possibility is that assessment and/or participation in a clinical trial per se may actually effectuate behavioural change. The assessment process may result in improved insight and awareness of depressive symptoms, and thus initiate coping mechanisms or help-seeking behaviour [75]. Improvements in non-intervention control groups have also been attributed to the increased attention or information provided as part of data collection (e.g., the Hawthorne effect; see 77). Therapeutic effects of trial participation itself would explain the overall symptom improvement observed within our sample, and the finding of steepest rates of recovery for the most severely depressed at baseline, as any therapeutic benefit is likely to be greatest for participants who are symptomatic to begin with. Given this study was conducted in a low-income area in Chile, we speculate that mental health literacy may have been low in this group, and thus the provision of basic symptom assessment may be particularly advantageous. The potential benefits of trial participation have received considerable attention in the substance abuse literature, with a recent meta-analysis finding improved outcomes (ranging from 11.5% to 46% symptom reduction) for control group participants in 15 of the 16 general population studies examined [78]. However, in the absence of corroborative outcome data it is unclear whether this reflects real behavioural change, or changes to symptom reporting. 

The present finding of decreasing depression symptom trajectories irrespective of trial allocation has far-reaching implications. If trial participation per se is indeed associated with beneficial effects, then simple symptom monitoring may prove a relatively inexpensive strategy for minimising the severity and long-term consequences of adolescent depression. However, the possibility that these improvements reflect retesting effects points to the importance of alternate assessment strategies, such as multiple informant reports and behavioural tests, in order to corroborate self-reporting of symptoms. For clinical trials research, the possibility of socially desirable or reactive reporting in control groups is a serious concern, as this may undermine generalisability to real-life contexts and reduce the sensitivity of studies to detect between group treatment effects. It is interesting to note that the observed decline in depression symptom trajectories over time was more pronounced for girls as compared to boys. This may suggest that girls are more sensitive to retesting effects, or more likely to benefit from symptom monitoring. However, at this stage it is not clear whether this pattern of results is broadly generalisable, or specific to the cultural context of the current study (Chile). Greater understanding of the effects of repeated assessment is clearly important, in order that methodological recommendations be made to minimise threats to internal validity. 

### Anxiety as a Precursor for Depression

Although we found an overall growth pattern of decreasing symptoms over time, there was inter-individual variability around this trajectory. Pre-existing anxiety has been identified as a risk factor for subsequent depression, and our findings replicated this pattern for girls but not boys. For girls, initial anxiety levels predicted slower recovery in depression over the course of the study. It is important to note that initial levels of depression did not influence subsequent anxiety, in support of the notion that anxiety is a precursor for depression rather than the other way around [21-23]. Several authors have argued that anxiety may be a particularly important developmental precursor for girls, contributing to the gender differences in depression that emerge during adolescence [25,30,31]. In a prior investigation, Chaplin et al. [31] showed that anxiety and worry symptoms were stronger predictors of subsequent depression for girls as compared to boys. Through the use of growth curve methodology, our findings extend these results, demonstrating that for girls, pre-existing anxiety also predicts the *rate of change* in depression symptoms. The observed gender differences suggest different developmental pathways for girls as compared to boys. A causal explanation of the relationship between anxiety and subsequent depression holds that anxious withdrawal and inhibition lead to social isolation and/or peer rejection, resulting in reduced self-esteem and, over time, symptoms of depression [31,79]. This developmental pathway may be particularly relevant for girls, who are more concerned about their peer relationships, and are more likely to derive self-worth from these relationships [80]. Alternate developmental pathways may be more important for boys, for example, there is some evidence that aggressive, self-aggrandizing boys are more likely to develop depressive symptoms [81]. On the other hand, we cannot rule out a non-causal explanation for our results, which may simply indicate that girls are more likely to experience genetic and/or environmental vulnerabilities common to both anxiety and depression. There is some epidemiological evidence suggesting that genetic factors play a greater role in the development of depression in women [82,83]. Thus, this internalising pathway from anxiety to depression may characterise an endogenous form of depression that is more common for girls. 

### Problem-Solving Ability as a Protective Factor

Contrary to previous work, we found no evidence that problem solving ability protects against the development of depressive symptoms. Within this cohort we observed an overall pattern of decreasing depressive symptoms over time, yet there was no evidence that problem-solving ability aided the recovery process. Previous studies have either evaluated this relationship cross-sectionally [34,36], or have demonstrated that baseline problem-solving ability predicts depressive symptoms at a later time point [32,39].To our knowledge, this study is the first to use growth curve methodology to explore the relationship between problem-solving ability and *rate of change* in depression symptoms. Thus, our study addresses different questions to prior longitudinal studies, which may explain the discrepancy in results. We did, however, find a cross-sectional association between problem-solving ability and lower initial levels of depression. Interestingly, we also observed a positive relationship suggesting that capacity for problem-solving was actually associated with higher levels of initial anxiety. This might suggest that the social-problem solving inventory confounds problem-solving style with cautiousness and anxious disposition. 

### Strengths and Limitations

Strengths of the current study include the longitudinal design, large sample size and relatively good questionnaire completion rates (82% and 77% at 6 and 18 month follow-up respectively). Although there is a body of research delineating risk factors for adolescent depression, these studies have typically been conducted in high-income Western countries. This study adds a novel perspective by extending the study of depression risk factors to a unique sample of socio-economically disadvantaged adolescents in Latin America. Furthermore, the latent growth analysis used in this paper has several advantages over traditional longitudinal analyses, as it allowed for explicit modelling of within-person changes in depression over time, and assessment of the impact of static and time-varying risk factors on symptom trajectories. 

As with any study, a number of limitations should also be considered. First and foremost, self-report measures are subject to demand characteristics, and as discussed previously may be susceptible to reactive retesting effects. Although we validated the BDI-II against clinical interview at baseline, it would have been ideal to incorporate multiple assessment strategies at all 3 time-points; however we note that this is rarely feasible in large samples. In the absence of alternate assessment sources, it is unclear whether the current results represent a true reduction in depressive symptoms over time, or simply changes to the way adolescents report their symptoms. Secondly, the age range of adolescents in this sample was quite large, which raises the possibility that adolescents at the extreme ends of the distribution may have differed in characteristics other than age (e.g. academic aptitude). For this reason, we conducted a sensitivity analysis in a sample restricted to adolescents aged 14 to 15 only (78% of the sample), which made no material difference to the pattern of results. Finally, our growth model was restricted to data collected on 3 measurement occasions, which precluded the possibility of testing time specifications other than linear.

### Implications for Research and Practice

A number of important implications for the study and treatment of adolescent depression are suggested by this research. Depression appears to be highly prevalent in this sample of Chilean adolescents, which may be a reflection of the lower socio-economic status within this sample. Our findings lend support to the hypothesis that anxiety symptoms are precursors to depression for girls but not boys. For clinicians, this points to the prognostic value of anxiety in girls as an early warning sign, and the potential benefit of early intervention to prevent subsequent development of depression. In addition, the surprising finding of decreasing depressive symptoms over the course of this study raises important clinical and methodological questions. Clinically, it would be useful to explore the potential benefits of brief depression interventions involving basic information and symptom assessment, akin to those commonly used to motivate behavioural change (e.g., primary care interventions to moderate alcohol intake; see 84). Brief interventions of this kind may be particularly beneficial in low socio-economic areas where mental health literacy is low, and costs need to be kept to a minimum. However, without further investigation firm conclusions regarding the potential benefits of assessment per se cannot be drawn, as it is unclear whether the observed pattern of recovery represents true change, or the effects of repeated testing. This study draws attention to the potential impact of regression to the mean and reactive retesting effects, which each represent a serious methodological concern for any clinical trial or longitudinal study. Given the potential threats to internal validity, future research should aim to systematically examine these retesting effects to determine more specifically why and under what conditions they occur. 
